# Membrane Stress and Ferroptosis: Lipid Dynamics in Cancer

**DOI:** 10.3390/ijms27020690

**Published:** 2026-01-09

**Authors:** Jaewang Lee, Youngin Seo, Jong-Lyel Roh

**Affiliations:** 1 Department of Otorhinolaryngology-Head and Neck Surgery, CHA Bundang Medical Center, CHA University, Seongnam 13496, Republic of Korea; 2 Logsynk, Seoul 06164, Republic of Korea; 3 College of Medicine, Gyeongsang National University, Jinju 52709, Republic of Korea; 4 Department of Biomedical Science, General Graduate School, CHA University, Pocheon 11160, Republic of Korea

**Keywords:** aldehydes, cholesterol, EMT, lipid peroxidation, pH, plasma membrane

## Abstract

Membrane rupture, induced by lipid peroxidation, is a severe threat to osmotic balance, as membrane pores contribute to ferroptosis, an iron-dependent cell death. To alleviate osmotic stress, membrane constituents dynamically reconstruct the membrane and interact with intracellular molecules. Tumor-derived acidosis shift glycolysis-dependent metabolism toward lipid metabolism, increasing polyunsaturated fatty acids (PUFAs). PUFAs enhance membrane fluidity but make cancer susceptible to lipid peroxidation. Also, the ionization of phospholipids under low pH can accelerate membrane rupture. This stress can be mitigated by the redistribution of cholesterol, which maintains tension–compression balance and acts as antioxidants. When excessive reactive aldehydes—byproducts of lipid peroxidation—overwhelm cholesterol’s protective role, lipid peroxides promote membrane cracks. Moreover, a deficiency in glutathione can alter cholesterol’s function, turning it into a pro-oxidant. In contrast, ceramide, derived from membrane lipids, indirectly prevents ferroptosis by facilitating cytochrome c release. This review integrates recent findings on how membrane components and environmental stressors influence ferroptosis. It also suggests potential therapeutic strategies. This could advance our understanding of ferroptosis in cancer.

## 1. Introduction

Lipids provide energy, form membranes, and function as signaling molecules. Lipids can be broadly classified into simple, complex, and derived lipids, and they can be further subdivided into more specific categories. However, since the structure of lipid classes is complex, please refer to another relevant paper for more detailed information [1]. Until recently, asymmetric structures of lipid bilayers—the fact that cells maintain a seemingly unstable structure—have been an unsolved issue. Despite its asymmetric structure, the plasma membrane effectively maintains osmotic pressure, protecting cells from external or internal threats. The balance between resting tension in the cytosolic leaflet and compression in the exoplasmic leaflet stabilizes the asymmetrical structure, using ATP-driven enzymes and cholesterol (Chol) redistribution [2,3]. Excessive phospholipids (PLs) in the inner leaflet increase resting tension and hydrophobic defect while transiently exposing the hydrophobic center to water. Redistributions of Chol can prevent membrane instability induced by excessive PLs or reducing sphingomyelin, which increases lipid droplets (LDs) [3,4]. Conversely, Chol depletion causes PL rebalancing and exposes phosphatidylserine (PS), resulting in apoptosis [5,6]. To prevent a devastating situation, the ER-associated GRAM domain-containing 1B (GRAMD1b) senses Chol imbalance and promotes redistribution independently of Chol biosynthesis [7,8]. Interestingly, Chol has contradictory functions. Chol oxidation propagates lipid peroxides via double bonds and the vinyl methylene group in the B-ring [9]. However, Chol derivates, including 27-hydroxycholesterol (27HC) and 7-dehydrocholesterol (7-DHC), trap LPO [10,11,12]. The dual nature of Chol accentuates the importance of regulating Chol in the redox system. Notwithstanding the well-documented antioxidant properties of Chol, the reason why metastatic cancer prefers Chol uptake to synthesize it is unclear.

Moreover, the plasma membrane is easily affected by fatty acyl chains of PLs. Polyunsaturated fatty acids (PUFAs), one of the representatives of fatty acyl chains and a key factor of lipid peroxidation (LPO), promote LPO and subsequently lead to ferroptosis, an iron-dependent cell death [13,14,15], while monounsaturated fatty acids (MUFAs) block it [16]. During ferroptosis, aldehydes, a product of LPO, elevate membrane permeability by forming adducts and functioning as zwitterions under specific conditions, further promoting LPO [17]. Subsequently, LPO exacerbates membrane rupture and tension while depressing Na^+^/K^+^-ATPase, activating piezo-type mechanosensitive ion channel component 1 (Piezo 1) and the transient receptor potential (TRP) channel [18]. This change dissipates monovalent cation gradients, thereby boosting ferroptosis under a ferroptosis inducer. In this context, many anti-ferroptosis molecules have been revealed, including glutathione peroxidase 4 (GPX4) [19], ferroptosis suppressor protein 1 (FSP-1) [20,21], GTP cyclohydrolase-1 (GCH1)/tetrahydrobiopterin (BH_4_) [22], coenzyme Q (CoQ) [23], dihydroorotate dehydrogenase (DHODH) [24], and aldehyde dehydrogenase 7 family member A1 (ALDH7A1) [25]. The anti-ferroptosis mechanisms of these molecules are based on their role as antioxidants. Although anti-ferroptotic molecules effectively suppress ferroptosis, metastatic cancer often counters them by increasing PUFAs. This strategy represents a trade-off, as PUFAs enhance cellular motility but simultaneously heighten susceptibility to ferroptosis. This indicates that there may be another potential benefit. Given that PUFA levels rise under cold conditions, their advantage may be linked to energy provision.

As the environment changes cellular homeostasis, we need to pay attention to its effect on ferroptosis. Considering that tumor-derived acidosis is a general trait in cancer, pH is a key factor in cellular homeostasis [26]. Acidosis can disrupt the immune system, promote the Fenton reaction-driven LPO, and weaken cell to cell adhesion, thereby promoting invasiveness and mobility [27,28,29,30]. An acidic pH reduces the plasma membrane’s surface and increases its permeability, heightening osmotic pressure [31,32]. In addition, acidosis inhibits acyl-coenzyme A:cholesterol O-acyltransferase 1 (ACAT1) activity, preventing cholesterol esterification and accumulating free cholesterol in macrophages, leading to cell death [33].

Several reviews on ferroptosis have highlighted its history, redox mechanism, and lipid metabolism [34,35,36]. This has improved our understanding of the mechanisms underlying ferroptosis, but it limits insights into the roles and functions of membrane components in response to environmental stressors. Thus, we aim to avoid redundancy by focusing on specifically mechanistic insight from the dynamics of membrane components and their relevance to EMT in cancer. We elucidate the mechanisms through which the cell membrane shifts toward a ferroptosis-preferred state and the roles of its components in responding to environmental stimuli. Further, we delineate unresolved mechanistic aspects and suggest hypotheses to explain these uncertainties, considering the roles of cell membrane constituents. Finally, we discuss the potential implications of ferroptosis for cancer therapy and outline research directions to elucidate these in future studies.

## 2. Ferroptosis and Lipid Peroxidation

LPO mainly occurs in enzyme-dependent and enzyme-independent pathways [37]. LPO has three stages: initiation, propagation, and termination. The Fenton reaction facilitates enzyme-independent pathways. On the other hand, enzyme-dependent pathways are promoted by lipoxygenases (LOXs) [13], cytochrome P450 oxidoreductase (POR) [38,39], or cytochrome *b*5 reductase (CYB5R1) [38]. Among LOXs, arachidonate lipoxygenase 15 (ALOX15) interacts with phosphatidylethanolamine-binding protein 1 (PEBP1) to produce lipid peroxides [40,41]. The cystine/glutamate antiporter (xCT; SLC7A11) is a key molecule in ferroptosis. SLC7A11 imports cystine and maintains the GSH/GPX4 redox system [42]. BRCA1-associated protein-1 (BAP1), a tumor suppressor protein, deubiquitinates SLC7A11 promoter, thereby reducing SLC7A11 expression. This limits cystine uptake and promotes ferroptosis [43]. APC membrane recruitment protein 1 (AMER1), another tumor suppressor, facilitates ferroptosis by degrading both SLC7A11 and ferritin light chain in colorectal cancer cells [44]. Lysine methyl transferase 2B (MLL4) deficiency promotes ferroptosis by reducing ALOX12, ALOX12B, and ALOXE3 while increasing SLC7A11 and GPX4 [45]. Mutations in the RAS family are frequently observed in human cancers. Kirsten rat sarcoma virus oncogene homolog (KRAS)-mutant cancer cells increase transferrin receptor 1 and SLC7A11 expression [46,47,48], highlighting the link between KRAS mutation and ferroptosis.

Acyl-CoA synthetase long-chain family member 4 (ACSL4) promotes the esterification of PUFAs to acyl-CoA, especially adrenic acid (C22:4) and arachidonic acid (AA; C20:4) [49]. Oxidized arachidonoyl-PE (AA-PE-OOH) and adrenoyl-PE (AdA-PE-OOH) are reincorporated into membranes through lysophosphatidylcholine acyltransferases (LPCATs), elevating LPO [50,51]. By contrast, MUFAs, such as oleic acid (OA) and palmitoleic acid (POA), protect against ferroptosis [16]. However, there is no precise mechanism for how MUFAs repress ferroptosis.

## 3. Acidic pH

### 3.1. Acidity and the Plasma Membrane

**Figure 1:**
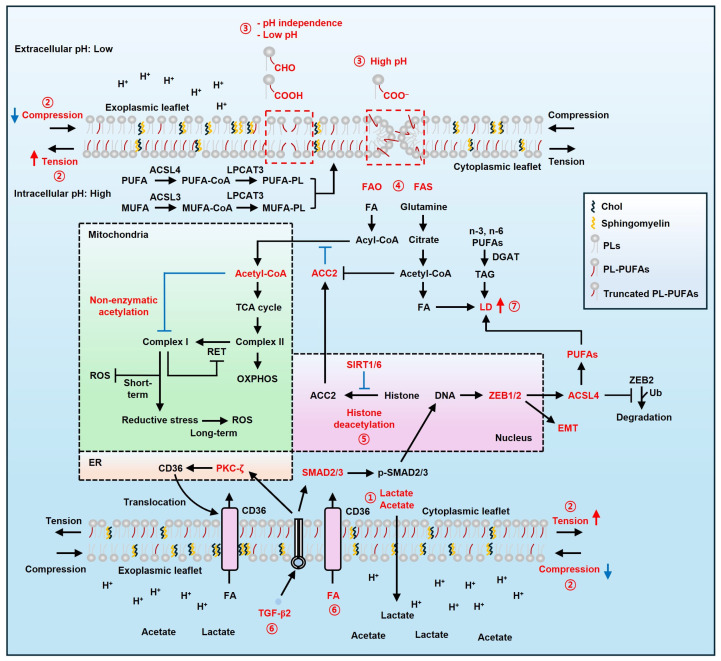
Changes in PM and cell metabolism at acidic pH. ① Cancer lowers the pH in the extracellular space by releasing acidic compounds such as lactate and acetate. ② This increases the vulnerability of the PM to the osmotic burden by elevating PM tension and permeability. ③ Aldehydes are unaffected by pH, while carboxylic acids react differently to pH levels. At acidic pH, both groups can form a structure that allows large molecules to pass into the cell. In contrast, small molecules pass through the pore formed by the carboxylate at high pH. ④ Additionally, acidic pH activates FAO, FAS, and glutamine metabolism. Increased acetyl-CoA inhibits ACC2 and complex I. ⑤ Furthermore, SIRT1/6 represses ACC2 transcription through histone deacetylation. ⑥ Moreover, acidic pH promotes EMT and fatty acid uptake via the TGF-β2/ZEBs axis and PKC-ζ, respectively. ⑦ These processes lead to the accumulation of LDs that support energy and antioxidation during EMT. Red accentuations mean the key molecules or events. See the Abbreviations Section for the terms.

A recent study explored how truncated oxidized phospholipids (OxPLs) affect membrane stability [52]. Oxidized phosphatidylcholines (OxPCs) influence lipid bilayer permeability based on pH and functional groups like aldehydes and carboxylic acids [53,54]. Δ9 OxPCs with aldehydes at *sn*-2 (16:0/9:0<CHO@C9) increase permeability regardless of pH, likely due to enhanced hydrophilicity (Figure 1) [52]. On the contrary, OxPCs with carboxylic acids (16:0/9:0<COOH@C9) depend on pH. At pH ≥ 7.5, ionized carboxyl groups promote membrane curvature, inducing toroidal pores and transient instabilities. At pH 4.5, non-ionized carboxyl groups and *sn*-2 acyl chains inhibit curvature and aggregation by protruding their chains to the membrane core. Aldehyde and non-ionized carboxyl groups facilitate larger molecule passage, while ionized carboxylate groups favor small hydrophilic molecules. Notably, FAs with aldehydes have low Gibbs free energy (∆G) [55,56]. This trait increases diffusion of small hydrophilic molecules, such as H_2_O, H_2_O_2_, and O_2_, across the bilayer, promoting the risk of fatty acid oxidation (FAO). Taken together, tumor acidosis may enhance membrane permeability, potentially making tumors more vulnerable to LPO.

### 3.2. Acidity and Lipid Metabolism

An acidic environment reprograms fatty acid (FA) metabolism in cancer cells via histone acetylation and non-enzymatic acetylation of mitochondrial complex I [57]. Under acidic conditions, FA and glutamine metabolism generate acetyl-CoA instead of glycolysis [57,58]. This change may occur to prevent an extremely acidic environment that cancer cells cannot tolerate, although the exact reason remains unclear. At an acidic pH, FA uptake increases without changes in FA transporter expression. Moreover, histone deacetylation by NAD-dependent deacetylase sirtuin 1/6 (SIRT1/6) represses acetyl-CoA carboxylase 2 (ACC2), while acetyl-CoA carboxylase 1 (ACC1) remains unaffected [57]. Although the exact mechanism that activates SIRT1/6 under acidic pH is unclear, an elevated NAD^+^/NADH ratio may contribute to their activation, as intracellular alkaline pH increases this ratio [59]. This change maintains a balance between FAO and fatty acid synthesis (FAS). Concomitant implementation of FAO and FAS may increase energy metabolism efficiency, supplying acetyl-CoA to the tricarboxylic acid (TCA) cycle. However, excess acetyl-CoA inhibits complex I activity through non-enzymatic acetylation. This prevents mitochondrial overfeeding and reverse electron transfer (RET) resulting from electron flow to complex II or CoQ imbalance (Figure 1) [60,61]. Nevertheless, prolonged inhibition of complex I may induce reductive stress [62,63], which can lead to ferroptosis [64,65,66]. Ultimately, acidic pH regulates ACC2 and lipid metabolism, reducing LPO and influencing cell survival. In parallel, acetyl-CoA may affect p53-mediated SLC7A11 regulation. p53 increases ferroptosis sensitivity by suppressing SLC7A11 transcription in lung cancer cells [67]. p53^4KR^ (K98/117/161/162R), prevents ferroptosis, though p53^3KR^ (K117/161,162R) still induces ferroptosis [68]. This implies that acetyl-CoA produced by acidosis may boost ferroptosis sensitivity in p53^4KR^ mutation.

As mentioned above, an acidic environment increases intracellular FAs, which can lead to LD accumulation [69]. Interestingly, acidic cancer cells preferentially accumulate LDs containing *n*-3 and *n*-6 PUFAs [70]. Under acidic conditions, *n*-3 and *n*-6 PUFAs promote lipid peroxidation when treated with erastin and RSL3, which are ferroptosis inducers. Inhibiting diacylglycerol acyltransferases (DGATs), which form triglycerides, prevents the formation of LDs and promotes ferroptosis in cancer cells exposed to acidic environments (Figure 1) [70]. Activation of DGAT1 protects cells against impaired lipid metabolism under iron depletion while promoting mitophagy and LD formation [71]. Conversely, DGAT1 inhibition hinders the formation of LDs and lysosome function, leading to endoplasmic reticulum (ER) stress and mitochondrial dysfunction, which in turn induces oxidative stress [72]. However, the role of LDs in mitophagy remains unclear. Given that LDs mitigate lipotoxicity from excessive FAs, they likely reduce LPO associated with iron deficiency, which triggers the iron starvation response and ferritinophagy [73,74]. Taken together, acidic pH can increase sensitivity to LPO in a DGAT-dependent manner [75,76].

Meanwhile, tumor acidosis activates the zinc finger E-box-binding homeobox 1 (ZEB1)/transforming growth factor beta (TGF-β2) pathway, promoting partial epithelial–mesenchymal transition (EMT) (Figure 1). This change accelerates the accumulation of LDs, enabling cancer cells to meet their energy demands through lipid metabolism. TGF-β2 also stimulates protein kinase C zeta (PKC-ζ)-mediated translocation of cluster of differentiation 36 (CD36), increasing the uptake of FAs for energy production via β-oxidation. Notably, ZEB1-induced EMT enhances PUFA uptake, thereby increasing membrane fluidity. This renders cancer cells vulnerable to lipid peroxidation (LPO); however, they utilize LDs to protect against lipotoxicity during metastasis, thereby supporting anoikis resistance and promoting invasiveness [77]. Thus, LD inhibition or lipophagy-mediated degradation can enhance LPO by releasing PUFAs [70,78].

## 4. Cholesterols

### 4.1. Physicochemical Trait of Cholesterol as a Potential Enhancer for LPO

**Figure 2:**
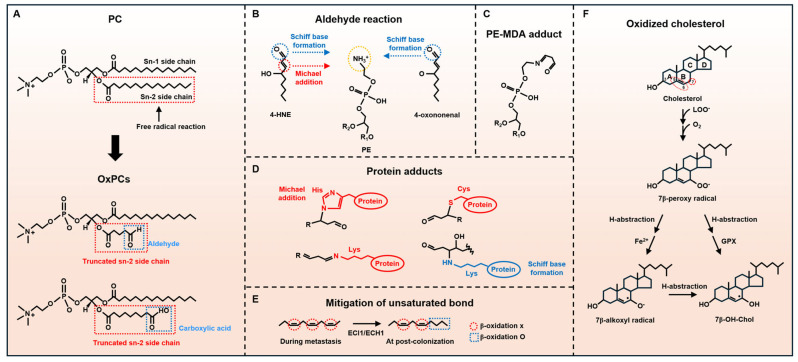
Lipids destabilizing PM, the alleviation process of unsaturated bonds, and the Chol oxidation process. (**A**) PC includes two fatty acid chains. The Sn-1 side chain is mainly saturated fatty acids. Unsaturated fatty acids mainly bind to the Sn-2 side chain, which is a prime target of ROS. ROS can truncate the Sn-2 side chain, forming OxPCs bearing aldehydes or carboxylic acids. Truncated OxPCs affect PM structure. (**B**–**D**) Aldehydes can form adducts via either Schiff base formation or Michael addition reaction to the amine group in proteins or PE. This destabilizes the PM. (**E**) Changes in double bonds at each step: metastasis and post-colonization. ECI1/ECH1 alleviates double bonds to fuel growth via β-oxidation. (**F**) Chol oxidation process. Chol can produce Chol radicals and may propagate lipid peroxidation. 4-HNE, 4-hydroxy-trans-2-nonenal; Cys, cysteine; ECI1/ECH1, enoyl-CoA delta isomerase 1/enoyl-CoA hydratase 1; GPX, glutathione peroxidase; His, histidine; LOO^●^, lipid hydroperoxyl radical intermediate; Lys, lysine; MDA, malondialdehyde; PC, phosphatidylcholine.

Chol is vital in eukaryotic cells, making up nearly 50% of the plasma membrane lipids and forming a low-permeability barrier against reactive oxygen species (ROS) [3]. Chol is positively related to cancer mortality [79]. It accumulates in exoplasmic plasma membrane leaflets and interacts with saturated lipids and sphingolipids [3]. Its unique structure, including the 5,6-double bond and vinylic methylene group at C-7, enables ROS trapping but also makes Chol vulnerable to oxidation when antioxidants are insufficient (Figure 2F). Hydroxyl radicals (OH^●^) and peroxynitrite (ONOO^●^) generate C-7-centered radicals in the B-ring via abstraction of C-7 with a weak carbon-hydrogen bond, propagating the free radical reaction [80]. Chol-hydroperoxides can cross cellular compartments and deplete glutathione (GSH), exacerbating oxidative stress [80,81,82]. Unlike plasma Chol, cellular Chol is highly prone to oxidation, preceding phospholipid oxidation in Jurkat cells under selenium depletion and butylated hydroxytoluene (BHT) exposure [83]. Considering that GPX4 is a selenoprotein, the role of Chol in redox balance—either as an antioxidant or a pro-oxidant—may rely on GPX4 levels in the context of ferroptosis. Conversely, plasmalogen phospholipids mitigate Chol peroxidation via their vinyl ether bonds [84,85]. Chol oxidation propagates radicals within lipid bilayers, suggesting its role in LPO under low antioxidant conditions, potentially influencing cancer progression and ferroptosis susceptibility.

### 4.2. 7-Dehydrocholesterol

**Figure 3:**
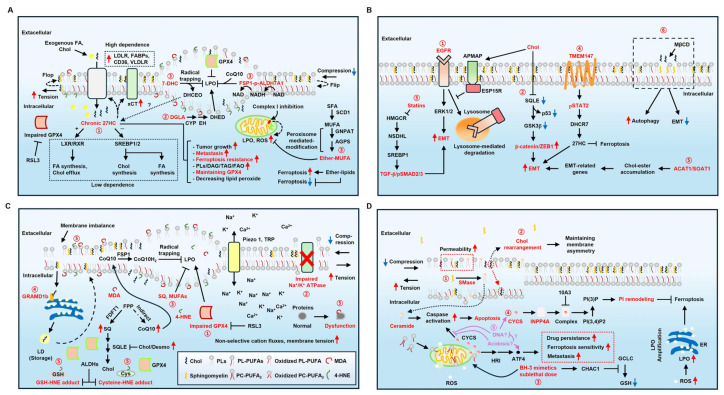
The role of PM components and derivates in ferroptosis and EMT. (**A**) ① Chronic 27HC increases cancer resistance to ferroptosis by reducing dependence on the mevalonate pathway and preserving GPX4 expression and activity. ② Supplementing DGLA promotes LPO, ③ which 7-DHC, ALDH7A1, and ether-MUFAs can suppress. (**B**) EMT can be promoted by Chol-related pathways. ① Chols stabilize EGFR signaling by increasing APMAP, which snatches ESP15R from EGFR. This inhibits EGFR degradation. ② Also, the increased influx of exogeneous Chols promotes β-catenin/ZEB1 axis by decreasing GSK3β and p53. ③ Statins activate TGF β/pSMAD2/3 axis. ④ TMEM147 activates pSTAT2 and increases 27HC. ⑤ ACAT1/SOAT1 increases EMT-related genes by accumulating Chol-ester. These pathways stimulate EMT and increase ferroptosis sensitivity. ⑥ In contrast, MβCD inhibits EMT and promotes autophagy. (**C**) ① Impaired GPX4 due to RSL3 disrupts PM integrity by increasing LPO. ② PM rupture intensifies membrane tension through uncontrolled cation fluxes, further aggravating PM rupture. ③ MUFAs, SQ, and CoQ can inhibit this destructive process. ④ Chols released from PM rupture are recognized by GRAMD1b in the ER and stored in LDs to recycle and reduce oxidative stress. ⑤ MDA and 4-HNE form PL adducts or protein adducts, causing membrane imbalance. (**D**) ① SMase increases PM permeability through the formation of ceramide, leading to apoptosis. ② Elevated PM stress results in Chol rearrangement to stabilize the PM, likely mitigating membrane rupture. ③ Meanwhile, BH-3 mimetics can reduce GSH and facilitate CYCS release. ④ CYCS interacts with INPP4A to prevent ferroptosis. ⑤ Purple accentuations indicate hypothetical pathways. Cytosolic DNA may contribute to the formation of the CYCS-INPP4 complex by inhibiting apoptosome assembly. Moreover, acidosis may increase ferroptosis sensitivity by activating ATF4 and suppressing apoptosis. Red accentuations mean the key molecules or events. See the Abbreviations Section for the terms.

Smith–Lemli–Opitz syndrome (SLOS) is a representative disease associated with 7-DHC accumulation [86]. The relationship between 7-DHC and ferroptosis has been recently elucidated. 7-DHC is a natural suppressor of ferroptosis [10], attributed to its conjugated double bond in the sterol B-ring, which serves as a strong H-atom donor to fatty acid peroxyl radicals [87]. This prevents oxidative damage while forming 3β,5α-dihydroxycholest-7-en-6-one (DHCEO) (Figure 3A) [88]. However, its protective effect diminishes under cholesterol supplementation, as free cholesterol inhibits sterol regulatory element-binding protein 2 (SREBP2) and the mevalonate pathway, leading to 7-DHC depletion. Inhibiting 7-dehydrocholesterol reductase (DHCR7), which converts 7-DHC into cholesterol via the Kandutsch–Russell pathway, prevents ferroptosis by accumulating 7-DHC [89]. Double knockout of DHCR7 and SC5D abolishes ferroptosis resistance. Supplementing 7-DHC effectively protects cancer cells from ferroptosis [90]. Among sterol analogs, only 7-DHC and ergosterol effectively block ferroptosis [11]. Increasing 7-DHC reduces ferroptosis in various cancer cells when combined with RSL3, AY9944 (EBP inhibitor), or cariprazine (DHCR7 inhibitor). However, SNU-1 and U937 cells, defective for 7-DHC biosynthesis, show minimal response to RSL3 plus AY9944. Supplementing 7-DHC effectively protects cancer cells from ferroptosis both in vitro and in vivo xenograft models, implying that inhibition of 7-DHC is closely related to potential therapeutic relevance.

### 4.3. 27-Hydroxycholesterol

27HC is known to promote EMT in breast cancer by stimulating immune cells toward immunosuppression [91]. Recent studies have demonstrated a correlation between 27HC and ferroptosis both in vitro and in vivo. Chronic exposure to 5 uM 27HC for 1–4 months, followed by maintenance with 1 μM 27HC, confers ferroptosis resistance in ER-negative breast cancer and melanoma cells by enhancing lipid uptake (Figure 3A) [12]. In contrast, acute exposure to 5 μM 27HC for 24–72 h disrupts SREBPs and liver X receptor (LXR)/retinoid X receptor (RXR) signaling, impairing the mevalonate pathway, which is crucial for GPX4 synthesis [92]. In 27HC-sensitive cancers, GPX4 levels decrease, whereas 27HC-resistant cancers maintain the mevalonate pathway, preserving GPX4 expression and activity, and increase xCT and lipid uptake through elevated lipid transporters (VLDLR, FABP4, CD36), thereby promoting tumor growth, EMT, and anti-LPO effects (Figure 3A). This shows that chronic 27HC enables cancer cells to depend less on de novo lipid synthesis and instead rely more on uptake mechanisms during tumorigenesis and metastasis under lipid-replete conditions. However, the reason why exposure to 27HC influences GPX4 expression remains unclear. Meanwhile, considering that 27HC is an oxysterol, whether DHCEO, as an oxysterol, can function in the same way as 27HC remains unclear. Transmembrane protein 147 (TMEM147) upregulates DHCR7 via signal transducer and activator of transcription 2 (STAT2) in hepatocellular carcinoma (HCC), elevating 27HC and GPX4, which strengthens ferroptosis resistance and metastasis (Figure 3B) [93]. HCC-derived 27HC boosts lipid metabolism and triggers peroxisome proliferator-activated receptor-γ (PPARγ) signaling in macrophages, leading to M2 macrophage polarization and enhancing HCC metastasis. Conversely, 27HC increases ROS-induced ER stress in leukemic cells, triggering apoptosis [94]. Overall, 27HC reduces ferroptosis susceptibility but promotes apoptosis in a cell type-dependent manner. Thus, targeting 27HC may represent an effective cancer therapy strategy in the context of ferroptosis.

### 4.4. CoQ and SQ

CoQ and SQ suppress ferroptosis by reducing LPO (Figure 3C) [21,90]. Sun et al. corroborated their role in cholesterol-mediated ferroptosis resistance [95]. Desmosterol (Desmo), Chol, and 7-DHC mitigate ferroptosis under RSL3 without altering the expression of ACSL4, GPX4, glutamate-cysteine ligase modifier subunit (GCLM), xCT, heme oxygenase (HMOX), DHODH, and NAD(P)H quinone dehydrogenase 1 (NQO1). Instead, Chol and Desmo enhance ferroptosis resistance via the FSP1-CoQ axis, effectively eliminating LPO [23]. Avasimibe, an ACAT inhibitor, increases resistance to RSL3 and cysteine deprivation, whereas methyl-β-cyclodextrin (MβCD), which extracts Chol from the plasma membrane, heightens sensitivity. Chol and Desmo promote squalene epoxidase (SQLE) degradation [96], increasing CoQ and SQ levels by redirecting carbon flux toward CoQ biosynthesis and reducing Chol synthesis. While farnesyl-diphosphate farnesyltransferase 1 (FDFT1) inactivation has little effect on ferroptosis, inhibiting both CoQ and SQ biosynthesis abolishes Chol’s protective impact. This highlights the interplay between cholesterol metabolism and ferroptosis suppression. Meanwhile, a recent study showed that p53 can lead to ferroptosis in human melanoma A375 cells independently of the GSH/GPX4 cycle by decreasing vitamin K epoxide reductase complex 1 like 1 (VKORC1L1). This prevents the vitamin K redox cycle and increases LPO [97].

### 4.5. Cholesterol and EMT

The relationship between EMT and cholesterol is intriguing. Oral squamous carcinoma cells (OSCCs) undergoing EMT exhibit low intracellular Chol levels [98]. Chol depletion in the plasma membrane enhances autophagy and suppresses EMT in cancer cells, concurrently inhibiting caspase-8 and triggering an undefined cell death pathway, likely ferroptosis [99,100,101]. Interestingly, exogeneous high Chol induces EMT through the extracellular-regulated protein kinases 1/2 (ERK1/2) pathway mediated by the inhibition of EGFR substrate 15-related protein (ESP15R) [102]. Adipocyte plasma membrane-associated protein (APMAP) interacts with ESP15R and activates the EGFR-ERK1/2 pathway, thereby initiating EMT in prostate cancer cells. Sterol O-acyltransferases 1 (SOAT1) [103], suppression of NAD(P)-dependent steroid dehydrogenase-like (NSDHL), and statins [104] also promote EMT by disturbing cholesterol metabolism in HCC and pancreatic mouse models with Kras^G12D^ expression and homozygous Trp53 loss. Impaired cholesterol metabolism results in the upregulation of LDLR expression for Chol uptake [105]. Mechanistically, colorectal cancer favors low Chol biosynthesis [106], whereas exogenous Chol promotes aggressiveness through SQLE inhibition and β-catenin/ZEB1 activation [107]. SQLE inhibition disrupts the GSK3β–p53 complex, preventing β-catenin degradation. As noted in Section 3.2, EMT can increase the risk of LPO via PUFAs. Besides PUFAs, iron may contribute to LPO under Chol depletion. Considering iron can induce Chol synthesis [108,109], Chol depletion may trigger ferritinophagy or transferrin receptor (TfR) expression to restore Chol levels, albeit at the cost of increasing ferroptosis risk. [15,110]. This implies that Chol-mediated EMT is a key determinant of ferroptosis sensitivity (Figure 3B). However, the reason why cancer does not directly synthesize Chol is still unknown. We propose two possibilities. First, given the role of Chol as a potential oxidant, reduced Chol metabolism may help prevent excessive ROS, with membrane receptors regulating cellular Chol levels instead. Second, an alternative hypothesis may involve energy conservation, as Chol biosynthesis is an ATP-intensive process. [111]. This metabolic demand can interfere with cellular activities such as motility, invasion, and extracellular matrix degradation during EMT, as these processes also require substantial ATP [112,113]. Consequently, cancer cells may decrease energy futility by reducing Chol synthesis.

## 5. Phospholipids

### 5.1. Fatty Acyl Tails

PLs are essential components of the plasma membrane that maintain cell survival. PLs are often truncated by ROS. Truncated PLs primarily contribute to membrane pore formation rather than the production of phospholipid hydroperoxides (PLOOHs) [55,114]. While PLOOHs stabilize membranes and preserve chemical gradients [55,115], truncated-chain PLs bearing aldehyde groups decrease the energy barrier for water permeation [55] and facilitate transmembrane pore formation by attracting water molecules into the lipid bilayer. In contrast, truncated lipids bearing carboxylic acid depend on pH.

PLs are classified by their head groups: phosphatidylcholine (PC), Phosphatidylethanolamine (PE), phosphatidylserine (PS), phosphatidylinositol (PI), phosphatidic acid (PA), and cardiolipin (CL) [116]. Each PL consists of two FAs at the *sn*-1 and *sn*-2 positions and a different phosphate headgroup at the *sn*-3 position of the glycerol backbone. The sn-1 position typically contains a saturated fatty acyl (SFA) tail, whereas the *sn*-2 position may include an SFA, a monounsaturated fatty acyl (MUFA) tail, or a polyunsaturated fatty acyl (PUFA) tail (Figure 2A). Since PUFAs cannot be synthesized de novo, mesenchymal cancers regulate the ratio of PLs with PUFAs and MUFAs by upregulating ACSL4, fatty acid desaturase 2 (FADS2), and ELOVL fatty acid elongase 5 (ELOVL5). Conversely, MUFA synthesis decreases through the downregulation of both stearoyl-CoA desaturase (SCD) and fatty acid synthase (FASN) [117]. This balance in PL composition affects membrane properties and cancer cell metabolism.

PUFAs are less abundant in the outer leaflet of the plasma membrane [118] but provide flexibility during metastasis. They promote ferroptosis via bisallylic hydrogens, which have a low energy barrier for H atom abstraction, favoring radical-mediated autoxidation and singlet oxygen (^1^O_2_) interactions [119]. Cancer cells with impaired redox system decrease PUFA incorporation into PLs as a defense mechanism against ROS [70,120]. Moreover, exposure to oxidized lipids increases lipid peroxidation [121]. PLs with two PUFA tails, though rare, drive ferroptosis more effectively than mixed acyl PLs [122]. Under RSL3 treatment, OAs (C18:1) completely block ferroptosis induced by monoacyl-PUFA phosphatidylcholines (PC-PUFA_1_s) but only partially inhibit ferroptosis from diacyl-PUFA phosphatidylcholines (PC-PUFA_2_s), implying PC-PUFA_2_s likely contribute to the downstream of PUFA incorporation. PC-PUFA_2_s disrupt mitochondrial complex I, integrate into the mitochondrial membrane, and render it vulnerable to superoxide production [60]. Mitochondrial ROS subsequently amplify LPO in the ER [123], ultimately triggering ferroptosis [124].

Interestingly, LPO can be constrained by endogenous ether lipids, which are mainly found in the cytosolic leaflet of the plasma membrane and function as antioxidants [125]. However, ether lipids can also increase ferroptosis sensitivity by releasing excessive PUFAs from triglyceride (TG) under lipid depletion [126]. Subsequently, PUFAs are incorporated into phospholipids, including ether forms. This supports cellular processes but increases sensitivity to ferroptosis. A recent study shows that dihomogamma-linolenic acid (DGLA, 20:3*n*-6) can also induce ferroptosis in *Caenorhabditis elegans* (*C. elegans*) and HT-1080 cells by converting DGLA into dihydroxyeicosadienoic acids (DHEDs) through the action of CYP-EH (CYP, cytochrome P450; EH, epoxide hydrolase) (Figure 3A) [125,127]. Meanwhile, p53 can protect cancer cells against ferroptosis by upregulating calcium-independent phospholipase A2β (iPL2β). iPL2β cleaves acyl tails of PLs and subsequently reduces oxidized PLs. This decreases LPO and enhances resistance to ferroptosis [128]. Moreover, p53 promotes the p21-GSH axis or deactivate dipeptidylpeptidase 4 (DPP4), both of which inhibit ferroptosis [129,130]. However, the mechanisms and conditions under which p53 induces ferroptosis remain to be elucidated.

In contrast, MUFAs protect against lipid peroxidation after colonization, enabling safe growth. Under hypoxic and nutrient-deficient conditions, cancer cells primarily produce MUFAs, helping cancer cells to evade ferroptosis [117]. Similarly, KRAS mutation upregulates ACSL3 and increases MUFA-PLs in lung cancer cells [16,131]. Mutant KRAS increases fatty acid synthase (FASN), promoting SFA and MUFA synthesis [132]. These traits stabilize redox system and protect the plasma membrane from LPO, ultimately enhancing ferroptosis resistance. While their precise anti-ferroptotic mechanism is unclear, studies suggest MUFAs interfere with PUFA synthesis [122], and MUFA-linked ether phospholipids (O-C18:1) reduce mitochondrial ROS in pancreatic ductal adenocarcinoma (PDAC) (Figure 3A) [133]. Another possibility involves MUFAs integrating into plasmalogen, where the vinyl ether bond, with low dissociation energy, acts as a sacrificial ROS trap, thereby delaying oxidation and limiting LPO [134,135,136,137,138].

Meanwhile, the monocyte-to-macrophage differentiation factor (MMD), a Golgi scaffold protein, directly interacts with ACSL4 and lysophospholipid acyltransferase 7 (MBOAT7) in OVCAR-8 and 786-O cells. This interaction forms AA-PI and other AA-containing phospholipid species, increasing ferroptosis sensitivity [139]. Taken together, PUFA-MUFA interactions shape ferroptosis susceptibility and impact cancer cell survival.

### 5.2. SMase, CYSC, and PI3P

Sphingomyelinase (SMase) is primarily located in the lysosome and extracellular space [140]. OxPC activates SMase, which converts sphingomyelin into ceramide and phosphocholine (Figure 3D) [3,140,141]. This change increases membrane permeability, destabilizing membrane structures. OxPC induces mitochondrial dysfunction [142] and ceramide activates caspases [143]. Thus, OxPC and ceramide may be able to promote ferroptosis or apoptosis by promoting LPO or activation of caspase in a context-dependent manner [141]. Mitochondrial dysfunction is closely linked to cytochrome c release. Cytosolic cytochrome c (CYCS) appears to have a dual role in cell survival. CYCS suppresses ferroptosis by interacting with inositol polyphosphate-4-phosphatase type I A (INPP4A) (Figure 3D) [144]. The CYCS-INNP4A complex increases phosphatidylinositol-3-phosphate (PI3P) production, which blocks phospholipid peroxidation and the plasma membrane rupture. Compound 10A3, blocking CYCS-INPP4A interaction, increases ferroptosis sensitivity in MEFs and PANC1 cells, stimulating immunostimulatory effects in vivo.

In contrast, the drug-persister cancer cell induced by a sublethal BH-3 mimetic exhibits a different tendency. A sublethal BH-3 mimetic increases CYCS by disrupting mitochondria. CYCS-eIF2AK1/heme-regulated inhibitor (HRI)-ATF4 axis leads to cell survival and metastasis instead of inducing apoptosis. However, this allows the drug-persister cancer cells to be susceptible to ferroptosis due to decreased GSH levels. This is achieved by increased ChaC GSH-specific gamma-glutamylcyclotransferase 1 (CHAC1) and repression of glutamate-cysteine ligase catalytic subunit (GCLC) (Figure 3D) [145]. Consequently, this increases sensitivity to ferroptosis.

Nevertheless, two questions remain unresolved: First, the reason why CYCS does not form an apoptosome in the cytosol under ferroptosis induction remains unclear. Interestingly, a recent study demonstrated that cytosolic DNA and CYCS compete for binding to apoptotic protease activating factor-1 (Apaf-1). The DNA-Apaf-1 complex initiates an inflammatory response instead of apoptosis [146]. Moreover, given that mitochondrial damage leads to the release of mtDNA and a reduction in ATP production [147], which is required to form apoptosome [148], the decrease in ATP and the released mtDNA may hinder assembly of apoptosome, thereby triggering inflammation. This leads to the hypothesis that this process may enhance the propensity of CYCS to associate with INPP4A rather than participate in apoptosome assembly. Second, another unresolved question remains regarding what determines whether CYCS binds to INPP4A or HRI. Notably, some studies showed that acidic pH can disrupt ATP depletion-induced apoptosis by preventing the interaction between caspase-9 and Apaf-1 [149] and activate ATF4 [150]. Furthermore, INPP4s exhibit optimal activity within the physiological range (pH 6.5–pH 8.0) [151,152]. This implies that under acidic conditions, CYCS preferentially interacts with ATF4 rather than associating with INPP4A when mitochondrial integrity is compromised. However, these hypotheses should be validated through future studies.

### 5.3. Reactive Aldehydes

Reactive aldehydes are formed by LPO and used as key markers of ferroptosis (Figure 2B). Representative examples include α,β-unsaturated aldehydes such as 4-hydroxynonenal (HNE) and acrolein; di-aldehydes such as malondialdehyde (MDA) and glyoxal; and keto-aldehydes such as 4-oxo-trans-2-nonenal (ONE) and isoketals (IsoK) [17]. PE is the second most abundant phospholipid following PC. Some reactive aldehydes target the amine group of PE, forming MDA-PE adduct, HNE-PE adduct, or ONE-PE adduct via Schiff base formation or Michael addition (Figure 2B–D) [17,153,154]. HNE can inactivate membrane-associated catalase in cancer cells, attenuating detoxification [155]. In addition, HNE binds GSH and cysteine, forming HNE–cysteine or HNE-GSH conjugates (Figure 3C). These processes consume GSH and compromise membrane integrity. Notably, proteins containing cysteine residues are highly susceptible to HNE, and the formation of HNE–protein conjugates impairs protein function and propagates oxidative stress [156]. This may exacerbate LPO-induced membrane rupture, ultimately leading to ferroptosis. Although GSH levels may subsequently recover [157], if the initial depletion is rapid or exceeds cellular restoration capacity, ferroptosis progression is likely irreversible.

To mitigate the cytotoxic effects of HNE, cancer cells employ physical elimination or enzymatic detoxification. Colon cancer releases HNE–cysteine into the extracellular space [158]. Also, reactive aldehydes are mainly detoxified by aldehyde dehydrogenases (ALDHs) [156,159]. Meanwhile, phospholipase D1 and D2 cannot hydrolyze N-aldehyde-modified phosphatidylethanolamines (NALPEs) generated by lipid peroxidation. NALPEs have cytotoxic and pro-inflammatory effects [153]. *N*-acyl phosphatidylethanolamine phospholipase D (NAPE-PLD) hydrolyzes NALPEs [160]. These mechanisms reduce ferroptosis risk by limiting LPO. Drawing on the chemical properties of aldehydes, researchers developed β-cyclodextrin-polyacryloylmorpholine (PCAM-βCD) to overcome the inherent instability of HNE. This compound has potent anticancer activity while exhibiting minimal toxicity in normal cells. In melanoma studies, it has shown promising therapeutic potential [161].

### 5.4. PUFAs and EMT

ACSL4 and PUFAs promote metastatic extravasation and colonization in ovarian cancer [162]. Although the sources of PUFAs remain unclear, adipose tissue and the transcription factors ZEB1 and ZEB2 likely supply cancer cells with PUFAs [163,164,165]. During EMT, cancer cells become more flexible due to increased PUFA content, which in turn heightens their dependence on GPX4 [166]. This implies that cancer cells undergoing EMT are more susceptible to ferroptosis when GPX4 is inhibited, thereby promoting LPO.

Although ZEBs do not directly regulate ferroptosis sensitivity, they contribute to ferroptosis-friendly cell conditions by increasing PUFAs and inducing EMT [164,167]. ZEB1 has been shown to enhance ferroptosis sensitivity through its role in EMT [164]. ZEB2 directly upregulates transcription of ACSL4 by directly binding to the ACSL4 promoter. ACSL4, in turn, protects ZEB2 from ubiquitin-mediated degradation by forming the ACSL4-ZEB2 complex (Figure 1). ACSL4 depletion reduces membrane fluidity, weakening invasion and extravasation during metastasis. Conversely, ACSL4 and PUFAs augment metastasis in cancer cells.

Significantly, ACSL4 may contribute to metastasis at the pro-colonization stage, but enoyl-CoA delta isomerase 1/enoyl-CoA hydratase 1 (ECI1/ECH1) may be required for cell growth at the post-colonization stage to fuel energy [168]. ECI1/ECH1 catalyzes the migration of C=C bonds of UFAs to resume the mitochondrial β-oxidation (Figure 2E) [169,170]. This process is essential because β-oxidation removes saturated and linear fatty acyl chains to produce acetyl-CoA. In the metastatic phase, increased PUFAs strengthen ferroptosis sensitivity under ferroptosis insults by promoting LPO.

## 6. Potential Therapeutic Applications

### 6.1. Radiotherapy

Resistance to chemotherapy and radiotherapy is a major challenge in cancer treatment. Resistant cancer cells exhibit a common trait, including enhanced lipid metabolism, drug efflux, and an improved DNA repair system. Notably, chemo- and radiotherapy-induced stress increases ROS, which promotes lipogenesis. Enhanced lipogenesis reduces membrane fluidity, permeability to anticancer drugs, and LPO. Interestingly, radiotherapy impairs SLC7A11 expression and cystine uptake in an ATM-dependent manner. Conversely, radiotherapy increases ACSL4 expression and PUFA-PLs, consequently elevating LPO and causing plasma membrane rupture [171,172]. Irradiated tumor cells can transfer microparticles (RT-MPs) to neighboring unirradiated cells through the radiation-induced bystander effect (RIBE). RT-MPs contribute to ROS generation and kill tumor cells via ferroptosis [173]. Although the mechanism by which RT-MPs induce ferroptosis remains unclear, radiation-mediated oxidized PUFAs may be transferred and propagate LPO. This behavior may represent an evolutionary strategy to help neighboring cells prepare antioxidant defenses, similar to how damaged mitochondria are transferred to other cells.

By contrast, radiotherapy can also increase SLC7A11 and GPX4 as an adaptive response, thereby boosting resistance to radiotherapy [171]. Targeting SLC7A11 or GPX4 can sensitize radioresistant cancer cells to ferroptosis [171]. Notably, the S phase of the cell cycle appears to be an optimal time to induce ferroptosis because radioresistant cancer cells significantly increase LDs lysis and FAO despite their enhanced resistance to radiotherapy [174,175]. Radioresistant cancer cells may be particularly vulnerable to LPO during this phase. Moreover, FAO produces acetyl-CoA, which is required for activation of the DNA damage sensor poly(ADP-ribose) polymerase 1 (PARP1) [176]. Ferroptosis inducers may further increase LPO since PARP1, with its low Km value, consumes NAD^+^, disrupting the antioxidant system and NADPH generation [177]. In addition, radiotherapy promotes pro-survival pathways such as PI3K–AKT, which are induced by PLs. This pathway promotes FAS and Chol synthesis to repair damaged membrane. In the nucleus, ATP citrate lyase (ACLY) provides acetyl-CoA for histone acetylation at double-strand breaks, increasing radioresistance in cancer cells [178]. Radioresistance induced by ACLY can be overcome using SB204990, an ACLY inhibitor. SB204990 promotes ferroptosis by ubiquitinating FSP1 [179].

### 6.2. Chemotherapy

Chemotherapeutic agents favor lipophilic interaction with the plasma membrane. To overcome their effects, the plasma membrane modifications appear to limit drug penetration and increase chemoresistance [180]. Upon exposure to chemotherapeutic agents, cancer cells activate HMG-CoA reductase (HMGCR), which elevates membrane Chol levels, contributing to membrane rigidity and altered permeability [181,182]. Combining HMGCR inhibitors with GPX4 inhibitors exerts anti-cancer effects in therapy-resistant cancer cells with a high-mesenchymal phenotype [183,184]. However, the use of HMGCR inhibitors remains controversial due to inconsistent efficacy in cancer prevention, warranting further research [185].

ATP-binding cassette (ABC) transporters act as drug efflux pumps and lipid floppases, translocating lipids from the inner to the outer leaflet of the plasma membrane. Lipid rearrangement induced by ABC transporters expels chemotherapeutic agents into the extracellular space, enhancing chemoresistance [186]. Exogenous PUFAs directly reduce the expression and function of P-glycoprotein (P-gp), an ABC transporter, in human colon cancer cells, thereby increasing intracellular accumulation of paclitaxel and 5-fluorouracil (5-FU) [187,188]. This implies that ferroptosis induction may exert a synergistic effect. Supporting this hypothesis, enhanced FA uptake via CD36 has been observed in human glioblastoma cells. Although increased FA uptake fuels FAO to produce NADPH and ATP, FAO inhibition with etomoxir, a CPT1 inhibitor, increases ROS and decreases ATP [189,190]. In this conditions, ferroptosis inducers may accelerate LPO, a mechanism potentially applicable to leukemia, ovarian, and pancreatic cancer cells, which exhibit resistance to cytarabine, doxorubicin, etoposide, irinotecan, gemcitabine, and cisplatin due to similar chemoresistant pathways [191,192,193].

Interestingly, chemoresistant cancer cells markedly overexpress GPX4 and SLC7A11 [194,195]. In parallel, malignant cancer cells reduce lipid saturation and the incorporation of PUFAs into membrane while increasing MUFA content in the plasma membrane [196,197], limiting drug entry. This indicates that combining ferroptosis inducers with PUFAs may help bypass resistance. Moreover, chemoresistant cells accumulate LDs to counter oxidative stress [198,199]. Although lipophagy could reduce LDs, lipophagy inducers are currently lacking. Compounds with potential lipophagy-inducing properties have been examined in clinical trials for non-alcoholic fatty liver disease (NAFLD) [200]. Cisplatin depletes GSH by forming cisplatin–GSH complexes [201,202]. GSH depletion can enhance LPO [203]. However, since cisplatin–GSH complexes account for only ~20% of GSH depletion [201], additional factors likely contribute. Paclitaxel can inhibit SLC7A11, further reducing GSH and increasing LPO [204]. In addition, malignant tumors and chemoresistant cells exhibit dysregulated iron metabolism, leading to elevated intracellular iron and ferritin heavy chain levels [195]. These findings imply that ferroptosis induction could overcome chemoresistance.

## 7. Conclusions and Perspectives

**Figure 4:**
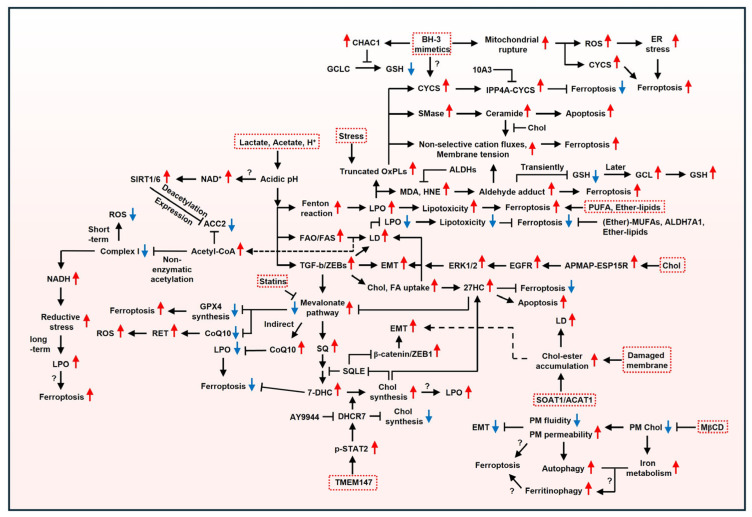
The schematic traffic map. This figure illustrates the relationship between stimuli and membrane components in ferroptosis. The red boxes, serving as starting points, guide cellular processes in response to stimuli. Please refer to the Abbreviations Section for the terms.

Overall, membrane plasticity plays a critical role in cancer cell survival in response to both external and internal stimuli (Figure 4). Here, based on physicochemical traits, we outline the influence of the plasma membrane constituents on cancer and molecular mechanisms activated when cancer cells are exposed to internal or external stressors. Subsequently, we link these processes to ferroptosis to broaden the understanding of the dynamic interaction between the plasma membrane remodeling and ferroptosis. If you want to learn about the interrelationships among multiple apoptosis pathways, please refer to another relevant paper [34,205]. Tumor acidosis is a defining characteristic of cancer. Tumor acidosis is a defining characteristic of cancer. Given that acidic pH increases membrane permeability, ferroptosis induction is likely to represent a promising strategy for cancer therapy, as ferroptosis fundamentally depends on the plasma membrane rupture. In particular, patient-derived cancer cells are likely to have different plasma membrane components. In this case, substituting carboxylates with aldehydes may increase vulnerability to ferroptosis by supplying aldehyde-enriched lipids. In addition, high extracellular Chol levels may reflect enhanced aggressiveness due to increased Chol release during EMT, suggesting its potential role as a biomarker for ferroptosis induction. Considering that aggressive cancers contain high PUFA levels to increase motility, ferroptosis induction may have a significant impact on cancer elimination. Drug-tolerant cancer cells pose a major challenge in cancer therapy. Interestingly, CYCS decreases GSH at the cost of survival under sublethal BH3 mimetics, implying that ferroptosis could serve as an alternative therapeutic approach in drug-tolerant cancers. Furthermore, numerous studies indicate that drug-persister cancer cells exhibit mesenchymal phenotypes and are vulnerable to ferroptosis, supporting the utility of ferroptosis induction in these cells. Understanding the dynamic interaction between the plasma membrane remodeling and ferroptosis may provide insights into unresolved questions that cannot be fully explained by internal factors or a single factor.

Nevertheless, several key questions remain unanswered:

(1) Unlike aldehydes, carboxylic acids form different structures under low and high pH. Considering that cells are usually cultured at neutral pH, the plasma membrane composition may influence ferroptosis induction. Can the ratio of phospholipids (PLs) with aldehydes to PLs with carboxylic acids in the plasma membrane affect ferroptosis induction?
(2) The abundance of PLs exacerbates membrane instability under low cholesterol (Chol). Can increasing PLs containing monounsaturated fatty acids (MUFAs) promote ferroptosis under low Chol conditions?
(3) The CYCS–INPP4A complex confers ferroptosis resistance by increasing phosphatidylinositol-3-phosphate (PI3P), which blocks lipid peroxidation (LPO). In contrast, CYCS can also increase ferroptosis sensitivity, though the precise mechanism remains unknown. Can PI3P function as a determinant in the type of cell death?
(4) Chol depletion promoted by methyl-β-cyclodextrin (MβCD) in the plasma membrane causes an undefined cell death while facilitating autophagy. Considering that cholesterol synthesis is related to iron metabolism, MβCD may promote ferritinophagy, a type of autophagy, and lead to ferroptosis. Future studies should investigate cell death in the context of ferroptosis.
(5) Ether lipids decrease ferroptosis sensitivity under normal conditions but increase it under lipid depletion. However, the mechanisms determining ferroptosis sensitivity remain unclear. Given that cholesterol contains a vinyl methylene group similar to ether lipids, the role of ether lipids in ferroptosis may be associated with cholesterol levels. Thus, future studies need to clarify these mechanisms and their relationship.

## Data Availability

No new data were created or analyzed in this study. Data sharing is not applicable to this article.

## Abbreviations

AA, arachidonic acid; ACAT1, acyl coenzyme A:cholesterol O-acyltransferase 1; ACC1/2, acetyl-CoA carboxylase 1/2; ACSL4, acyl-CoA synthetase long chain family member 4; ALDHs, aldehyde dehydrogenases; ALDH7A1, aldehyde dehydrogenase 7 family member A1; ALOX15, arachidonate lipoxygenases 15; Apaf-1, apoptotic protease activating factor-1; APMAP, adipocyte plasma membrane-associated protein; ATP, adenosine triphosphate; BHT; butylated hydroxytoluene; *C. elegans*, *Caenorhabditis elegans*; CD36, cluster of differentiation 36; CHAC1, ChaC Glutathione (GSH)-Specific Gamma-Glutamylcyclotransferase 1; Chol, cholesterol; CL, cardiolipin; CoQ, coenzyme Q; CYB5R1, cytochrome b5 reductase; CYSC, cytosolic cytochrome c; CYP-EH, cytochrome P450- epoxide hydrolase; Desmo, desmosterol; DGAT, diacylglycerol acyltransferases; DGLA, dihomogamma-linolenic acid; 7-DHC, 7-dehydrocholesterol; DHCEO, 3β,5α-dihydroxycholest-7-en-6-one; DHCR7, 7-dehydroChol reductase; DHED, dihydroxyeicosadienoic acids; DHODH, dihydroorotate dehydrogenase; ECI1/ECH1, enoyl-CoA delta isomerase 1/enoyl-CoA hydratase 1; EGFR, epidermal growth factor receptor; ELOVL5, ELOVL fatty acid elongase 5; EMT, epithelial–mesenchymal transition; ER, endoplasmic reticulum; ERK1/2, extracellular-regulated protein kinases 1/2; ESP15R, EGFR substrate 15-related protein; FA, fatty acid; FABP, fatty-acid-binding protein; FADS2, fatty acid desaturase 2; FAO, fatty acid oxidation; FAS, fatty acid synthesis; FASN, fatty acid synthase; FDFT1, farnesyl-diphosphate farnesyltransferase 1; FSP-1, ferroptosis suppressor protein 1; GCL, glutamate cysteine ligase; GCLC, glutamate-cysteine ligase catalytic subunit; GCLM, glutamate-cysteine ligase modifier subunit; GCH1/BH4, GTP cyclohydrolase-1/tetrahydrobiopterin; GSK3β, Glycogen Synthase Kinase-3 beta; GNPAT, glyceronephosphate O-acyltransferase; GPX4, glutathione peroxidase 4; GRAMD1b, GRAM domain containing 1B; GSH, glutathione; 27HC, 27-hydroxycholesterol; HCC, hepatocellular carcinoma; HMOX, heme oxygenase; 4-HNE, 4-Hydroxynonenal; INPP4A, inositol polyphosphate-4-phosphatase type I A; HRI, heme-regulated inhibitor; LD, lipid droplet; LDLR, low-density lipoprotein receptor; LOO^●^, lipid hydroperoxyl radical intermediate; LOX, lipoxygenase; LPCAT, lysophosphatidylcholine acyltransferase; LPO, lipid peroxidation; LXR, liver X receptor; MβCD, methyl-b-cyclodextrin; MBOAT7, lysophospholipid acyltransferase 7; MDA, malondialdehyde; MMD, monocyte-to-macrophage differentiation factor; mtDNA, mitochondrial DNA; MUFA, monounsaturated fatty acid; NAD, nicotinamide adenine dinucleotide; NADH, nicotinamide adenine dinucleotide (reduced form); NALPEs, N-aldehyde-modified phosphatidylethanolamines; NAPE-PLD, N-acyl phosphatidylethanolamine phospholipase D; NQO1, NAD(P)H quinone dehydrogenase 1; NSDHL, NAD(P)-dependent steroid dehydrogenase-like; OA, oleic acid; OxPCs, oxidized phosphatidylcholines; OH^●^, hydroxyl radicals; ONOO^●^, peroxynitrite; OxPLs, oxidized phospholipids; PA, phosphatidic acid; PC, phosphatidylcholine; PC-PUFA1s, monoacyl-PUFA phosphatidylcholines; PC-PUFA2s, diacyl-PUFA phosphatidylcholines; PDAC, pancreatic ductal adenocarcinoma; PE, phosphatidylethanolamine; PEBP1, phosphatidylethanolamine-binding protein 1; PI, phosphatidylinositol; Piezo 1, piezo-type mechanosensitive ion channel component 1; PI3P, phosphatidylinositol-3-phosphate; PKC-ζ, protein kinase C zeta; PL, phospholipid; PLOOH, phospholipid hydroperoxide; POA, palmitoleic acid; POR, cytochrome P450 reductase; PS, phosphatidylserine; PPARγ, proliferator-activated receptor-gamma; PUFA, polyunsaturated fatty acid; RET, reverse electron transport; ROS, reactive oxygen species; SCD, stearoyl-CoA desaturase; SC5D, lathosterol oxidase; SFA, saturated fatty acid; SIRT1/6, NAD-dependent deacetylase sirtuin 1/6; sEVs, small extracellular vesicles; SLOS, Smith-Lemli-Opitz syndrome; SMase, sphingomyelinase; SOAT1, Sterol O-acyltransferases 1; SQ, squalene; SQLE, squalene epoxidase; SREBP, sterol regulatory element-binding protein; STAT2, signal transducer and activator of transcription 2; TGs, triglycerides; TGF-β2, Transforming growth factor beta; TME, tumor microenvironment; TMEM147, transmembrane protein 147; TRP, transient receptor potential; TRP53, transformation-related protein 53; xCT, system xc−cystine/glutamate antiporter; ZEB1/2, Zinc finger E-box-binding homeobox 1/2.
